# Hedgehog Signalling in Osteogenesis and Bone Metabolism: Molecular Mechanisms, Regulatory Networks and Implications for Skeletal Disease

**DOI:** 10.1111/jcmm.70813

**Published:** 2025-08-26

**Authors:** Rohey Njie, Shihan Xu, Taofen Wu, Jiashun Pi, Sisi Lin, Pengxiang Zhang, Jiaqi Wang, Qi Dai, Hui Shen, Nenghua Zhang, Guiqian Chen

**Affiliations:** ^1^ Department of Biopharmaceutics, Zhejiang Provincial Engineering Research Center of New Technologies and Applications for Targeted Therapy of Major Diseases, College of Life Science and Medicine Zhejiang Sci‐Tech University Hangzhou China; ^2^ School of Life Sciences University of Liverpool Liverpool UK; ^3^ R&D Department Ceva Animal Health Technology (Hangzhou) Co. Ltd. Hangzhou China; ^4^ Clinical Laboratory Jiaxing Hospital of Traditional Chinese Medicine Jiaxing China

**Keywords:** BMP signalling, bone formation, *Hh* signalling, osteoblasts, TGF signalling, Wnt signalling

## Abstract

The Hedgehog (Hh) signalling pathway serves as a fundamental regulator in bone development and homeostasis, translating extracellular signals into precise transcriptional programmes that govern osteogenic differentiation and bone remodelling. Central to this process, ligand‐dependent Hh activation induces the nuclear translocation of GLI transcription factors (GLI1/2/3), which orchestrate the expression of key osteogenic regulators, including RUNX2 and Osterix (OSX), thereby directing mesenchymal stem cell (MSC) fate commitment. Among Hh ligands, the Indian hedgehog (Ihh) plays a dominant role in endochondral ossification, spatiotemporally controlling osteoprogenitor differentiation and chondrocyte maturation. Notably, the Hh pathway engages in extensive, context‐dependent crosstalk with Wnt/β‐catenin, BMP, TGF‐β, FGF and PTHrP signalling cascades, forming a highly interconnected regulatory network essential for skeletal patterning and morphogenesis. Dysregulation of this balanced system contributes to a spectrum of skeletal disorders, ranging from congenital defects to degenerative bone diseases, highlighting its critical role in maintaining bone integrity. This review synthesises recent advances in Hh‐mediated osteogenesis, dissecting its multi‐layered interactions within the skeletal gene regulatory framework. By unravelling the molecular logic of Hh‐dependent signalling networks, we deepen our understanding of bone biology and illuminate novel therapeutic targets for skeletal pathologies through precision modulation of Hh pathway activity.

## 1 Introduction

Bone is a highly dynamic and metabolically active tissue that continuously adapts to mechanical stimuli and repairs structural damage through tightly regulated processes, including remodelling. During skeletal development, coordinated bone modelling and remodelling shape skeletal architecture, maintain systemic mineral ion homeostasis and repair biomechanically compromised regions [1, 2, 3]. Beyond its mechanical role, bone is a protective organ, a haematopoietic niche and a critical regulator of calcium homeostasis [4]. In adults, bone integrity is maintained through a continuous remodelling cycle orchestrated by the balanced activity of osteoblasts (bone‐forming cells) and osteoclasts (bone‐resorbing cells) [5]. This process ensures the removal of damaged bone and its replacement with a new matrix, preserving mechanical function and mineral equilibrium [6]. Notably, neuropeptides secreted by bone‐innervating nerve fibres and resident bone cells fine‐tune osteoblast and osteoclast activity, adding another layer of regulation to skeletal homeostasis [7]. Osteoblasts are bone cells responsible for the formation of new bones and the growth and repair of old ones. They have a significant role in the pathophysiology of bones even though they comprise only 4%–5% of the total number of osteoprogenitors [8]. Other types of cells contribute to maintaining and repairing bones during homeostasis and these include osteocytes, osteoclasts and chondrocytes, wherein, osteoclasts are responsible for bone resorption, and osteocytes and chondrocytes contribute to bone development [9, 10, 11].

Bone development (osteogenesis) is a precisely orchestrated process whereby mesenchymal stem cells (MSCs) undergo lineage commitment to become either osteoblasts or chondrocytes, governed by an intricate network of signalling pathways including the evolutionarily conserved Hh pathway [12]. The Hh signalling pathway serves as a master regulator of bone development and remodelling, critically mediating the osteogenic differentiation of progenitor cells [13]. Two distinct but complementary mechanisms drive bone development: endochondral ossification, wherein MSCs first differentiate into chondrocytes to form a transient cartilaginous scaffold that is subsequently replaced by mineralised bone tissue [14, 15, 16]. This process underlies the development of most long bones, including weight‐bearing elements like the femur and tibia [17]. Intramembranous ossification, characterised by the direct differentiation of condensed MSCs into osteoblasts that deposit bone matrix without an intermediate cartilage template [18]. This mechanism generates flat bones such as those comprising the cranial vault [19] and clavicle [20].

Central to these processes is Indian Hedgehog (Ihh), which orchestrates the commitment of perichondrial mesenchymal cells to *Runx2* ^+^ osteoprogenitors, facilitating bone collar formation and primary spongiosa development [21, 22]. Runx2, the master regulator of osteoblast differentiation, is indispensable for both ossification programmes [21, 23]. The differentiation cascade further involves other key transcription factors, including Osterix (OSX), which drives the terminal differentiation of *Runx2* ^+^ progenitors into mature osteoblasts while simultaneously constraining their proliferative capacity [21], and *Drosophila* distal‐less 5 (DLX5), another critical determinant of osteogenic fate. However, the SRY‐box transcription factor 9 (SOX9) regulates chondrogenic commitment [9, 15]. Recent mechanistic insights reveal that Sonic hedgehog (Shh) activates *SOX9* expression through GLI1‐mediated regulation of distal chromatin interactions, establishing a direct molecular link between Hh signalling and chondrocyte specification [22].

The Hh pathway does not operate independently; instead, it engages in extensive crosstalk with other major signalling pathways, including Wnt/β‐catenin, BMP, PTHrP, FGF and TGF‐β to coordinate osteoblast differentiation and maturation [24]. Perturbations in this delicate signalling equilibrium—particularly dysregulation of Hh–Wnt interactions—contribute to various skeletal pathologies ranging from osteoporosis and osteopenia to osteoarthritis and osteosarcoma [21, 25, 26]. This review synthesises recent advances in our understanding of Hh signalling in skeletal biology, with particular emphasis on its complex interplay with other pathways in maintaining bone homeostasis. Furthermore, we explore emerging therapeutic strategies targeting Hh signalling components for treating bone‐related disorders, highlighting promising avenues for clinical translation.

### 1.1 The Hedgehog Signalling Pathway

The Hh signalling pathway represents one of the most evolutionarily conserved intercellular communication systems, maintaining remarkable functional fidelity from invertebrates to vertebrates. This sophisticated signalling pathway plays fundamental roles in multiple biological processes, including embryonic patterning, tissue homeostasis and osteogenic differentiation. Mechanistically, the pathway mediates precise transduction of extracellular signals from the cell membrane to the nucleus, enabling context‐dependent transcriptional regulation [24, 25]. The Hh signalling pathway plays essential roles in skeletal development by regulating osteoblast differentiation, chondrocyte proliferation and maturation, and precise skeletal patterning [27]. This evolutionarily conserved pathway derives its name from the hedgehog ligand initially identified in *Drosophila melanogaster* , where mutations caused a spiked embryonic phenotype resembling a hedgehog. In vertebrates, the Hh family comprises three structurally related ligands—Ihh, Desert hedgehog (Dhh) and Sonic hedgehog (Shh)—that serve as primary activators of this signalling cascade [28]. Among these, Shh and Ihh are particularly crucial for vertebral bone development, where they orchestrate cell differentiation programmes essential for proper bone and cartilage formation during vertebral growth [29]. In contrast, Dhh primarily functions in gonadal development and exhibits minimal involvement in osteogenic processes [30], highlighting the specialised roles of distinct Hh ligands in mammalian development.

The canonical Hh signalling pathway mediates its biological effects primarily through the activation of GLI transcription factors (GLI1, GLI2 and GLI3) that translocate to the nucleus to regulate the expression of target genes governing critical cellular processes, including proliferation, differentiation and maturation. At the membrane level, pathway activation is initiated when Hh ligands bind to the 12‐pass transmembrane receptor Patched (PTCH), thereby relieving its constitutive repression of the G protein‐coupled receptor Smoothened (SMO) and permitting SMO activation. This ligand‐receptor interaction triggers an intracellular signalling cascade that ultimately promotes nuclear accumulation of GLI transcription factors and subsequent transcriptional regulation of downstream targets [31, 32]. The GLI family members exhibit distinct functional properties, with GLI1 and GLI2 serving as primary transcriptional activators while GLI3 predominantly functions as a repressor, creating a sophisticated balance of activating and inhibiting signals that ensures precise modulation of pathway output [33].

**FIGURE 1:**
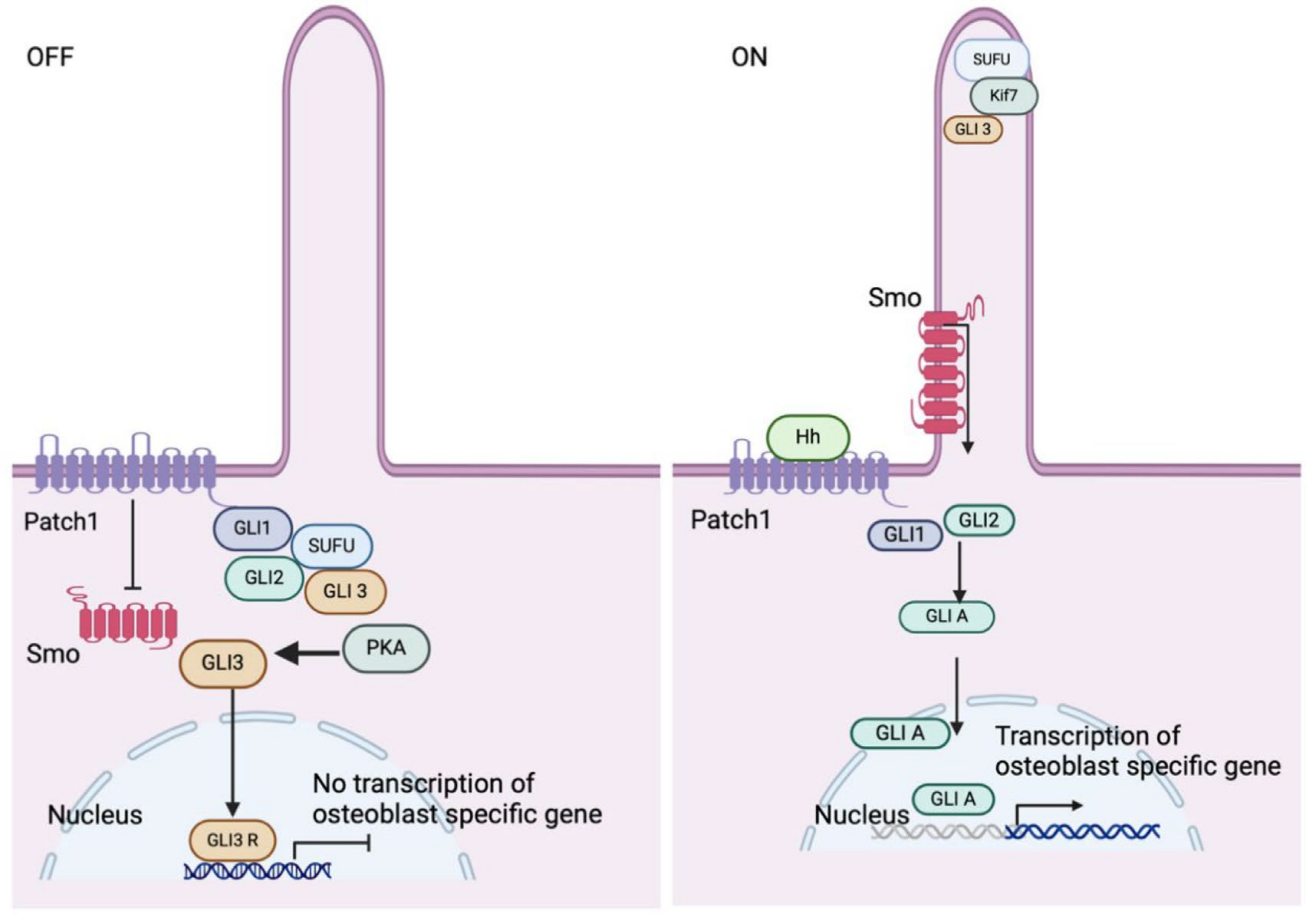
Canonical and non‐canonical Hedgehog signalling mechanisms in skeletal biology. In the basal state (Hh ligand absence), PTCH1 inhibits SMO, while SUFU and PKA bind and phosphorylate GLI transcription factors, generating GLI repressors (primarily GLI3R) that suppress osteogenic gene expression. Canonical pathway activation occurs when Hh ligands bind PTCH1, relieving SMO inhibition. Activated SMO promotes GLI activator formation (GLI1/2A) while Kif7 displaces SUFU from GLI3 at the primary cilium tip. The resulting GLIA translocates to the nucleus to induce osteoblast‐specific gene transcription, demonstrating the pathway's precise spatiotemporal regulation of osteogenesis.

Beyond the canonical GLI‐dependent signalling, Hh pathway activation can occur through non‐canonical mechanisms classified into two distinct modes: SMO‐dependent and SMO‐independent signalling [34]. In the basal state, SMO is maintained in an inactive conformation through PTCH‐mediated inhibition. At the same time, additional negative regulators including SUFU and PKA further suppress pathway activity by directly binding to and phosphorylating GLI transcription factors, respectively, to inhibit their transcriptional function [28, 35] (Figure 1). The dynamic equilibrium between canonical and non‐canonical Hh signalling is essential for appropriate cellular responses, as exemplified by intraflagellar transport protein 80 (IFT80), which orchestrates this balance during osteoblast differentiation by simultaneously promoting pro‐osteogenic canonical signalling while suppressing anti‐osteogenic non‐canonical Hh pathway activity [36]. This intricate regulatory network highlights the complexity of Hh signalling modulation, where the integration of multiple input signals and regulatory components enables precise control of cellular outcomes in a context‐dependent manner.

The Hh signalling pathway serves as a critical regulator of bone development and homeostasis, exhibiting stage‐specific effects during osteogenesis [37]. While Hh signalling promotes early osteoblast differentiation through upregulation of key transcription factors Runx2 and OSX, which is evidenced by impaired osteoblast proliferation upon *Smoothened (SMO)* deletion in MSCs [37, 38], its role becomes more complex during later stages of bone development. Mak et al. demonstrated that Hh signalling activity progressively decreases during osteoblast maturation, and notably, *SMO* deletion in mature osteoblasts unexpectedly increased bone mass and conferred protection against age‐related bone loss [25]. These seemingly paradoxical findings suggest a dual regulatory mechanism whereby Hh signalling promotes early osteogenic commitment while potentially suppressing terminal osteoblast differentiation.

Further highlighting the context‐dependent nature of Hh signalling, studies show that *Gli1*+ progenitor cells contribute to pathological heterotopic endochondral ossification by proliferating at post‐traumatic joint injury sites [39]. Mechanistic studies in mouse models revealed that guanine nucleotide‐binding protein, Alpha stimulating gene (GNAS) acts as a negative regulator of Hh signalling in progenitor cells through Protein Kinase A (PKA)‐mediated suppression, with GNAS mutations leading to enhanced Hh signalling activity that drives ectopic bone formation [40, 41, 42]. These collective findings not only elucidate the nuanced temporal regulation of osteogenesis by Hh signalling but also identify Hh pathway inhibition as a promising therapeutic strategy for preventing heterotopic ossification, particularly in trauma‐induced or genetic predisposition to ectopic bone formation.

### 1.2 Molecular Architecture of Hedgehog Signalling in Osteogenesis

#### 1.2.1 MSCs Commitment to Osteoblasts

Indian hedgehog (Ihh) emerges as a master regulator of skeletal morphogenesis. As the principal Hh ligand in bone development, Ihh initiates MSC specification into *Runx2* ^+^ osteoprogenitors, forming the foundation for bone development [24, 43]. At the molecular level, Ihh engages in reciprocal regulation with RUNX2/Cbfβ heterodimers and BMP/Wnt signalling pathways to drive osteogenesis [44, 45, 46, 47, 48]. Similarly, Sonic hedgehog (Shh) serves as a master regulator of bone development. It orchestrates MSC commitment by driving osteoblast differentiation from uncommitted MSCs via its action as a potent morphogen and through the activation downstream effectors including GLI1 and MAPK–ERK [49, 50, 51]. Shh also interacts with BMP and FGF8 pathways to direct ectoderm‐derived neural crest cells toward osteogenic fates [52, 53].

As the primary receptor of Hh ligands, Patched1 (PTCH1) acts as a gatekeeper of the pathway. Its expression in early osteoprogenitors positions it as a critical node linking ligand availability to downstream transcriptional activity. The PTCH1 receptor is a master regulator of bone development and homeostasis, with its dysfunction linked to profound developmental disorders [54] and metabolic bone diseases [55]. As the primary Hh pathway modulator, PTCH1 maintains precise control over osteoblast differentiation [56], which is evidenced by conditional knockout studies showing accelerated osteogenesis and increased bone mass upon *PTCH1* deletion [57]. Its critical role extends to bone marrow niche regulation, where *PTCH1* ablation disrupts haematopoietic progenitor differentiation while mobilising myeloid precursors [58]. These findings collectively position PTCH1 as both a crucial developmental regulator and a potential therapeutic target in bone biology.

Signal transduction proceeds through the G protein‐coupled receptor Smoothened (SMO), that bridges ligand‐dependent Hh signalling to GLI‐mediated nuclear transcription, driving early osteoblast specification via canonical GLI2‐mediated Runx2 expression [38, 59, 60, 61]. Regulation of SMO has been found important for limb development [62]. In stem cell niches, SMO acts as a fate‐determining switch, balancing self‐renewal and differentiation of mesenchymal progenitors [63, 64]. This positions SMO as a driver of osteoprogenitor differentiation. Downstream of SMO, the Glioma‐associated Oncogene Homolog (GLI) family, comprising GLI1 (activator), GLI2 (dual regulator) and GLI3 (repressor), serves as the final effectors of Hh signalling and orchestrates MSC commitment through a sophisticated balance of activator‐repressor dynamics, determined by developmental context and post‐translational modifications [25, 61, 65, 66, 67, 68, 69, 70, 71, 72, 73]. This functional versatility enables precise control of osteogenic programs, such as GLI2 directly regulating *Runx2* expression to drive osteoblast specification while modulating tissue homeostasis through its repressor activity [61]. Meanwhile, Suppressor of Fused (SUFU), the central negative regulator of Hh signalling, exerts multifaceted control over GLI proteins through cytoplasmic sequestration, promoting their PKA‐mediated cleavage into repressor forms (GLI‐R) while preventing nuclear translocation of GLI activators, effectively silencing target gene expression. This process ensures that osteogenic commitment occurs at appropriate developmental stage [74, 75, 76, 77, 78].

#### 1.2.2 Osteoblasts' Differentiation to Osteocytes

During osteoblast differentiation, SMO exhibits stage‐specific duality—driving early osteoblast proliferation and differentiation while restraining late‐stage maturation through PTHrP‐RANKL axis modulation [25, 38, 60]. This dual regulation of the pathway reflects a developmental switch that underscores its sophisticated adaptation to microenvironmental cues. Conditional deletion of *PTCH1*, a critical component of Hh signalling, accelerates osteoblast differentiation and increases bone mass [56, 57]. This finding positions PTCH1 as an inhibitor of terminal differentiation. Lineage‐tracing demonstrates that *GLI1+* progenitors give rise to osteoblasts and contribute to bone homeostasis during postnatal bone development [37, 79]. In addition, post‐translational modifications (acetylation, sumoylation, phosphorylation) modulate the transcriptional activity of GLI proteins, creating an effective timely transition of osteoblasts into osteocytes [80].

#### 1.2.3 Bone Resorption

Hh signalling enhances bone resorption by upregulating PTHrP‐RANKL expression via PKA and CREB [25]. Ihh signalling modulates bone formation and resorption to maintain skeletal homeostasis and biomechanical competence [81, 82]. Hh signalling activators (SMO, GLI1, GLI1/2) promote osteoclast differentiation, but only the SMO‐GLI1/2 axis targets both early and late‐stage differentiation, while GLI1 activation is only required in the early stage. Furthermore, *LysM‐Cre* models reveal that *SMO* deletion in the osteoclast lineage impairs osteoclast differentiation and bone resorption and paradoxically preserves bone mass during aging [60]. Similarly, *SUFU* ablation in myeloid lineages suppresses RANKL‐induced osteoclast differentiation and bone resorption via JNK/c‐Fos‐NFATc1 cascade repression by upregulated *Hh* signalling in vitro [83]. This highlights SUFU as a critical regulator of Hh signalling, suggesting that it prevents the excessive activation of Hh signalling in bone‐resorbing cells to maintain homeostasis.

#### 1.2.4 Intramembranous Ossification

In calvarial development, *Shh* promotes intramembranous ossification by regulating cranial neural crest dynamics while maintaining suture patency through BMP/MSX2‐mediated signalling [52, 84]. This balance suggests Shh likely reflects stage‐specific roles in intramembranous ossification, enhancing early mesenchymal proliferation and facilitating late‐stage osteoblast differentiation [85]. Furthermore, SUFU's regulation of GLI2 and GLI3 is essential in calvarial bone development. Its deletion in cranial neural crest cells enhances *GLI2* expression while depleting *GLI3*, causing calvarial defects rescued only by concurrent *GLI2* knockout [86]. Therapeutic applications leverage *Shh's* regenerative capacity in intramembranous bones: overexpression of an N‐terminal *Shh* peptide (*ShhN*) enhances bone repair by sustaining stem cell viability and scaffold integration [85], while Shh‐transduced cell therapies demonstrate significant cranial defect regeneration [87].

#### 1.2.5 Endochondral Ossification

During endochondral ossification, Ihh coordinates a precise developmental pathway. It drives perichondral osteoblast differentiation for bone collar formation while maintaining growth plate homeostasis, chondrocyte maturation through a PTHrP‐mediated negative feedback loop [27, 60, 66, 88, 89]. This feedback loop coordinates the spatiotemporal formation of the cartilage template [46, 66]. The GLI2/GLI3 axis orchestrates osteogenesis, with chondrocyte progression largely depending on GLI3 expression [70], whereas osteoblast differentiation necessitates its removal, and while GLI2 independently promotes hypertrophic cartilage vascularisation [47, 71, 72]. Genetic evidence highlights GLI2's indispensable role in endochondral ossification; its deletion triggers dysregulated chondrocyte proliferation while suppressing cartilage resorption and bone formation [90] (Table 1). This underscores GLI2's central position in balancing chondrogenic and osteogenic progression during endochondral ossification.

**TABLE 1:** Skeletal phenotypes associated with genetic alterations in Hedgehog signalling.

Gene	Cre	Phenotype	Refs.
*Shh*	*Hoxb6‐CreER*	Loss of hindlimb digits	[91]
*Shh*	*Col2a1*‐*Shh‐Cre*	Delayed chondrocyte differentiation and long bone defects/loss	[92]
*Ihh*	*Col2α1‐CreER*	Depletion of columnar structure, premature vascular invasion and ectopic hypertrophic chondrocytes formation of in the growth plate	[93]
*Ihh*	*Germline*	Osteoblasts fail to proliferate in endochondral bones	[89]
*Ihh*	*Prx1‐Cre*	Abnormal chondrocyte differentiation and osteogenesis.	[94]
*Ihh*	*Prx1‐Cre*	Growth plate and secondary ossification center deficiency	[88]
*Ihh*	*Col2alpha1‐Cre*	Delay in chondrocyte hypertrophy, malformed limbs	[44]
*Ihh*	*Col2a1‐Cre*	Decreased cartilage callus	[95]
*PTCH1*	*Mx‐Cre*	Loss of osteoblasts, increased *RANKL* expression, impaired bone formation	[57]
*PTCH1*	*Germline*	Leads to enhanced bone formation resulting in increased bone mass	[56]
*PTCH1*	*Rosa26CreERT2*	Block of T and B cell differentiation in the bone marrow	[58]
*SMO*	*Colα1(II)‐Cre*	Bone collar defects	[60]
*SMO*	*RosaCreER*	Reduced osteogenic differentiation and 50% reduction in periosteal bone callus formation	[38]
*SMO*	*Col1‐Cre*	Decreased amounts of bone tissue	[96]
*SMO*	*Sox9‐Cre*	Decreased cartilage callus formation and bone regeneration.	[97]
*SMO*	*LysM‐Cre*	Low trabecular bone mass	[98]
*GLI1*	*Gli1 CreERT2*	Decrease in bone mass	[37]
*GLI1*	*GLI1 + progenitors*	Promoting bone regeneration of the mandible	[79]
*GLI2*	*Germline*	Decrease in bone formation	[90]
*GLI3*	*Prx1‐Cre*	Smaller forelimb autopods	[99]
*SUFU*	*Wnt1‐Cre*	CNC‐derived calvarial bone loss	[100]
*SUFU*	*Dermo1‐Cre*	Mesoderm‐derived calvarial bone defects	[100]
*SUFU*	*SUFU* ^*OC*^‐Cre	Reduction in cellular cementum mass and a shorter root length	[101]
*SUFU*	*LysM‐Cre*	Enhanced bone formation	[83]

#### 1.2.6 Growth Plate Development

*Ihh* signalling orchestrates postnatal skeletal maintenance through dual regulation of bone architecture and repair mechanisms, as evidenced by *Col2α1‐CreER*‐mediated deletion of *Ihh*, revealing disrupted growth plate organisation, premature vascular invasion, ectopic chondrocyte hypertrophy and compromised trabecular bone integrity [93, 102]. Its mediation with PTHrP maintains the balance between chondrocyte proliferation and hypertrophy, ensuring longitudinal bone growth [46, 66]. Genetic evidence highlights the significant roles of Ihh signalling in the growth plate, with knockout models revealing skeletal defects such as growth plate disintegration and impaired long bone development [89, 94]. Transgenic mouse studies further elucidate *Shh's* developmental roles, where its overexpression disrupts growth plate organisation through elevated *SOX9* expression [92].

Beyond development, Shh emerges as a pivotal regulator of cartilage homeostasis and osteoarthritis (OA) pathogenesis, orchestrating complex cell–cell interactions within the articular microenvironment. In human osteoarthritic cartilage (OAC), Shh mediates crosstalk between MSCs and chondrocytes, driving pro‐inflammatory signalling pathways that promote tissue degeneration through mechanisms involving cellular senescence and inflammatory microenvironment establishment [103]. Paradoxically, Shh demonstrates chondroprotective potential in regenerative contexts, synergising with TGF‐β to enhance chondrogenesis in rabbit bone marrow stromal cells and initiating early‐stage cartilage repair processes, underscoring its fundamental involvement in cartilage morphogenesis [104].

#### 1.2.7 Bone Mineralisation

The Hh signalling pathway plays a crucial role in bone mineralisation by regulating osteogenic differentiation, chondrocyte maturation, intramembranous and endochondral ossification [27, 38]. Ihh influences the expression of *RUNX2* and *OSX*, thus promoting bone matrix mineralisation [105]. During fracture healing and regeneration, Ihh remains essential by activating periosteal progenitors and coordinating angiogenesis, which contributes to bone matrix mineralisation [48, 106]. Hh signalling also regulates connective tissue growth factor (CTGF), an osteoclast‐derived osteo‐inductive factor that promotes osteoblast differentiation and mineralisation [107]. Studies show that inhibition of Hh signalling by cyclopamine decreased bone mineralisation [108], while its overexpression via the Ihh/BMP‐2 axis enhanced mineralisation [109]. *GLI2* deficient mice decrease mineralised cartilage resorption, which can lead to excess mineralised tissue, resulting in skeletal defects [90]. The SUFU/GLI axis contributes to balanced Hh signalling at appropriate levels and duration, which is essential during mineralisation stages to prevent premature or excessive mineral disposition that can cause skeletal defects [75, 78, 100].

### 1.3 Hh Signalling in Crosstalk With Various Signalling Pathways in Osteogenesis

#### 1.3.1 Crosstalk Between Hh and Wnt/β‐Catenin Signalling Pathway

The Wnt/β‐catenin signalling pathway, also referred to as the canonical Wnt/β‐catenin signalling pathway, is a highly conserved pathway that contributes to skeletal development and tissue homeostasis by regulating osteogenesis [2]. The pathway activation involves the binding of Wnt/β‐catenin to its receptor complex (Frizzeled and its co‐receptor LRP5/LRP6) which leads to the stabilisation of β‐catenin and its translocation to the nucleus to promote the transcription of osteogenic genes [2, 110]. The Hh and Wnt/β‐catenin pathways engage in a sophisticated, stage‐specific dialogue that orchestrates skeletal development through reciprocal regulation of osteoblast and chondrocyte differentiation [2, 111]. At the core of this crosstalk, Ihh drives nuclear β‐catenin translocation and Wnt/β‐catenin target gene activation in the perichondrium [24], while GLI2 forms a positive feedback loop by enhancing β‐catenin transcriptional activity [112]. This synergistic interplay is modulated by shared nodal regulators—GSK3 and SUFU [113]—with SUFU acting as a brake on the system by suppressing Tcf‐dependent transcription [114, 115].

**FIGURE 2:**
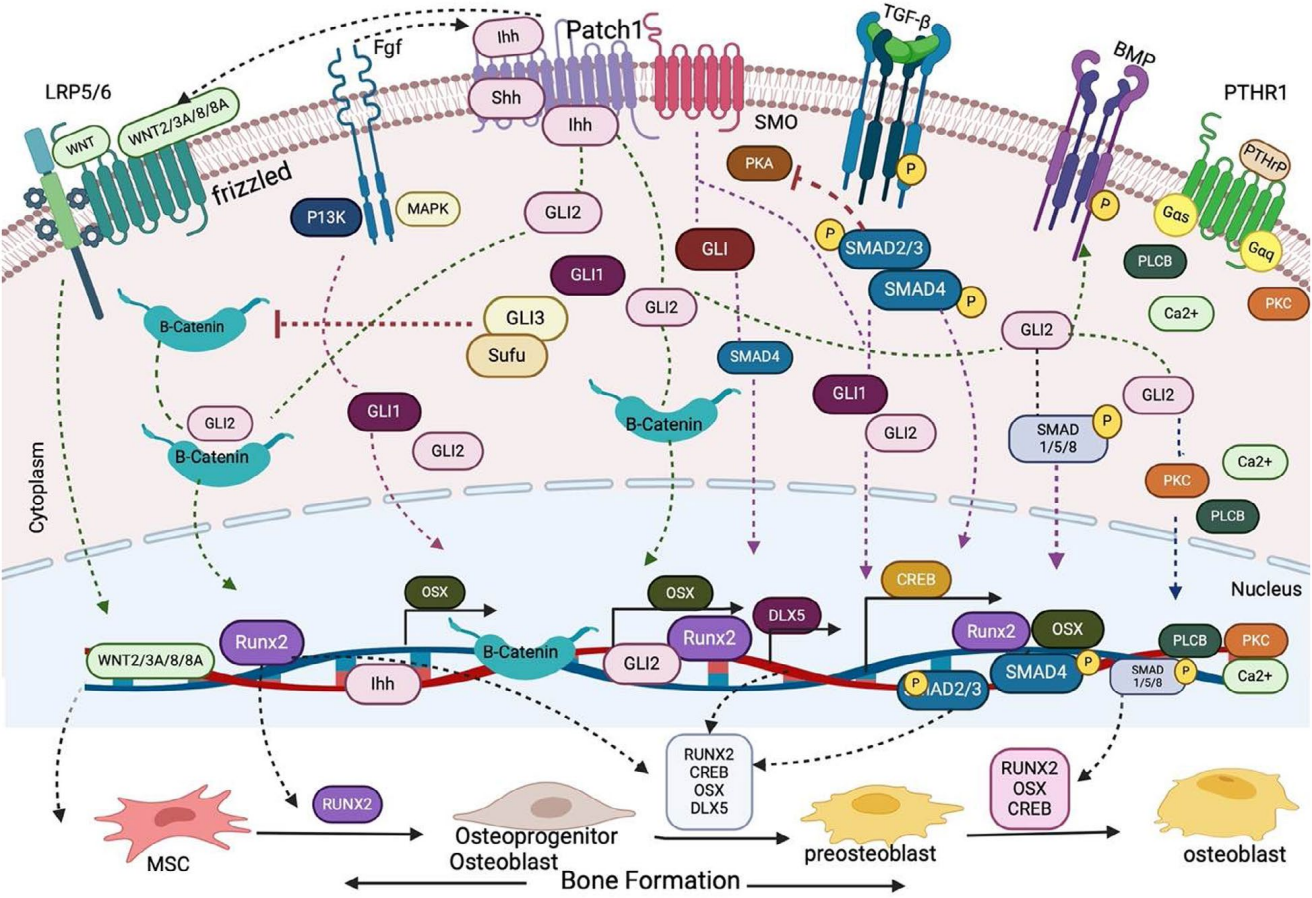
Hedgehog pathway crosstalk with other signalling pathways in osteogenesis. Hedgehog signalling orchestrates osteoblast differentiation through coordinated interactions with multiple pathways, forming an integrated regulatory network centred on key transcription factors. Ihh‐Wnt crosstalk stabilises β‐catenin to activate RUNX2 and OSX expression, while Shh‐FGF interaction amplifies osteogenic gene transcription (RUNX2/OSX/CREB). The Ihh‐PTHrP feedback loop maintains growth plate homeostasis by reciprocal regulation of these factors. TGF‐β signalling modulates this network through Smad4‐mediated suppression of GLI2 and Smad3‐dependent GLI1/2 activation via PKA inhibition, fine‐tuning Ihh/PTHrP balance. Concurrently, BMP‐Smad1/5/8 activation by GLI2 upregulates RUNX2, OSX and DLX5 to drive bone formation. This multilayered interaction converges on a core osteogenic programme where RUNX2 initiates mesenchymal commitment to osteoprogenitors, while cooperating with OSX and CREB to advance maturation—a late‐stage process progressively independent of Hh input, highlighting its stage‐specific dominance in early lineage specification versus diminishing role in terminal differentiation.

Genetic evidence reveals the pathway interdependence; *Ptch1* deletion in *Prx1+* MSCs triggers *Wnt5A/6* overexpression and *β‐catenin*‐driven skeletal defects [116], whereas *β‐catenin* loss in osteoprogenitors shunts differentiation toward chondrogenesis [117]. The pathways exhibit both cooperative and antagonistic behaviours, while Hh upregulates Wnt ligands (*Wnt2/3a/8/8b*) to promote osteogenesis [118], and *SMO* activation induces Wnt inhibitors (*Sostdc1/Dkk1*) that fine‐tune cementogenesis [101]. SOX9 emerges as a critical integrator, with its mutant form (*Y440X*) stabilising β‐catenin to modulate Ihh signalling in chondrocytes [119]. These interactions create a dynamic regulatory circuit where β‐catenin operates downstream of *Ihh* in osteoblasts yet upstream in chondrocytes [24, 114, 120, 121], with disruption causing severe morphogenetic defects like campomelia [119]. Collectively, these findings reveal an exquisitely balanced signalling network that spatially and temporally coordinates bone development through cell type‐specific modulation of Hh‐Wnt crosstalk (Figure 2).

#### 1.3.2 Crosstalk Between Hh and TGF‐β Signalling

The TGF‐β (Transforming Growth Factor‐β) signalling pathway plays a significant role in regulating osteogenic MSC differentiation, skeletal development and homeostasis. It transduces signals via Smads, leading to their translocation to the nucleus to regulate osteogenic gene expression [16, 122, 123]. The Hh and TGF‐β pathways form a reciprocal feedback loop that drives endochondral ossification through coordinated control of Ihh/PTHrP signalling and osteogenic commitment [16]. TGF‐β signalling amplifies *Ihh* expression, with *Smad4* deletion studies demonstrating its essential role in maintaining growth plate Ihh/PTHrP levels [94], while GLI‐mediated Hh activation reciprocally enhances TGF‐β production to reinforce osteoblast lineage specification [16]. This synergistic interplay is further modulated by Smad3 and β‐catenin, which collectively upregulate *GLI1/2* expression to potentiate osteogenic programmes—a mechanism hijacked in bone metastatic disease [124, 125]. TGF‐β fine‐tunes this network through PKA suppression, reducing phospho‐CREB levels and downstream *MITF* promoter activity to unleash *GLI* transcriptional output [126]. The precise balance of these cross‐activating signals creates a self‐reinforcing morphogenetic field that spatially and temporally coordinates chondrocyte progression and osteoblast differentiation during endochondral bone development (Figure 2), with pathway perturbations leading to either developmental defects or pathological ossification.

#### 1.3.3 Crosstalk Between Hh and BMP Signalling

The BMP (Bone Morphogenetic Protein) signalling pathway specifically via BMP2 and BMP4 drives osteogenesis through the Smad1/5/8 signalling by promoting [11, 127, 128]. The Hh and BMP pathways engage in a context‐dependent manner that orchestrates skeletal cell fate decisions, exhibiting synergistic and antagonistic interactions across spatiotemporal domains. While Ihh‐BMP cooperation drives osteoblast differentiation and bone collar formation [60], their relationship shifts dynamically across cellular compartments—GLI2‐mediated BMP2 activation by Shh promotes osteogenesis [16, 129], yet GLI activators paradoxically suppress BMP‐dependent osteoblast specification in perichondrial cells [24, 66, 130]. This yin‐yang regulation extends to chondrogenesis, where BMP operates independently of Hh signalling to maintain chondrocyte proliferation [131], yet requires Hh activity to prevent ectopic chondrocyte differentiation [23]. Epigenetic studies reveal another layer of control, with Hh ligands (Shh/Ihh/Dhh) modulating *BMP7* expression over osteogenic timelines [132], while GLI2 emerges as the central molecular integrator of this crosstalk [129]. These findings crystallise a dual regulatory paradigm: in osteogenesis, Hh and BMP function as cooperative partners driving differentiation, whereas in chondrogenic niches, Hh actively constrains BMP's osteogenic potential to preserve cartilage integrity, a delicate equilibrium whose disruption underlies both developmental and regenerative skeletal pathologies (Figure 2).

#### 1.3.4 Crosstalk Between Hh and FGF Signalling

The Fibroblast Growth Factor (FGF) signalling pathway is essential in skeletal development and homeostasis by regulating osteoblast lineage commitment, proliferation and differentiation. It also contributes to bone repair and regeneration, and it transmits its signals via the MAPK/ERK and PI3K/AKT pathways [117, 133]. The Hh and FGF pathways engage in a vertebrate‐specific feedback loop that precisely coordinates limb patterning and chondrogenesis. The Shh‐FGF10 axis forms a self‐regulating circuit where Shh signalling suppresses mesenchymal *FGF10* expression through the Hedgehog‐interacting protein (Hip1) [134], while FGFs reciprocally modulate chondrocyte behaviour by antagonising BMP‐mediated Ihh production [135]. This cross‐pathway regulation exhibits striking compartmental specificity, with FGFR3 operating upstream to inhibit Ihh signalling in growth plate chondrocytes [136] and FGF functioning downstream of Hh in anterior mesoderm patterning [137]. The inhibitory arm of this interaction reveals additional complexity, with FGF signalling directly suppressing GLI‐mediated transcription of *Shh* target genes [138]. However, Shh upregulates *FGF8* expression to promote MSC differentiation and survival of neural crest cells during cranial development [139, 140]. Together, these interactions create an adaptive signalling network where Hh and FGF pathways alternate as dominant regulators depending on developmental context, sometimes cooperating (as in limb bud outgrowth [141]), other times opposing (as in growth plate control [136]), to ensure precise spatiotemporal coordination of skeletal morphogenesis (Figure 2).

#### 1.3.5 Crosstalk Between Hh and PTHrP Signalling Pathway

Parathyroid hormone‐related protein (PTHrP) is one of the most important growth factors involved in regulating the proliferation and differentiation of chondrocytes in the growth plate while delaying their hypertrophic differentiation [142, 143, 144]. The Ihh‐PTHrP negative feedback loop emerges as a functionally critical interaction that orchestrates skeletal morphogenesis and coordinates chondrocyte progression in the growth plate while simultaneously directing osteoblast lineage commitment. This evolutionarily conserved mechanism begins with Ihh‐driven *PTHrP* upregulation, which maintains chondrocyte proliferation and delays hypertrophy through a precisely timed signalling cascade [131, 145], a process essential for both digit formation [94] and long bone elongation [146]. Ihh promotes RUNX2‐mediated osteoblast specification by suppressing GLI3‐repressor activity [147], while the RUNX2/Cbfβ heterodimer reciprocally amplifies *Ihh/PTHrP* expression through direct promoter binding [45], creating a self‐reinforcing loop. Beyond development, this axis extends its influence on bone remodelling, where Ihh‐induced PTHrP activates osteoclastogenesis via PKA‐CREB‐mediated *RANKL* production in osteoblasts [25, 148]. These findings collectively position the Ihh‐PTHrP circuit as a central signalling hub that integrates chondrogenesis, osteogenesis and bone homeostasis through stage‐specific modulation of transcriptional effectors and secondary messengers (Figure 2), offering multiple therapeutic entry points for skeletal disorders.

### 1.4 Molecular Pathogenesis of Skeletal Malformations Caused by Hedgehog Pathway Mutations

Genetic perturbations in Hh signalling components drive a striking array of skeletal malformations, revealing the pathway's exquisite sensitivity to dosage effects in orchestrating skeletal morphogenesis [149]. From neural tube patterning to limb development, Hh signalling exerts precise control over cellular processes [150]—evidenced by *Shh*‐null mice exhibiting characteristic distal limb truncations [151] and neural tube defects [152] that mirror human holoprosencephaly (HPE) with its concomitant craniofacial and skeletal abnormalities [153, 154]. *Ihh* deficiency manifests as profound limb shortening and calvarial hypoplasia [152], while more subtle *Ihh* mutations underlie clinical spectra ranging from brachydactyly type A1 to acrocapitofemoral dysplasia [155, 156]. The pathway's therapeutic vulnerability emerges through SPOP‐GLI3 regulatory axis manipulation, where *SPOP* depletion‐induced osteopenia and brachydactyly are rescued by GLI3 repressor attenuation [69]. These findings collectively demonstrate how Hh signalling operates as a molecular rheostat during bone development.

*Ptch1* mutations disrupt *Hh* signalling homeostasis, driving both neoplastic transformation and skeletal pathologies through pathway hyperactivation. As the primary Hh receptor, *Ptch1* loss‐of‐function mutations autonomously activate both canonical and non‐canonical signalling, manifesting clinically as Gorlin syndrome (characterised by BCCs and medulloblastomas) [157] and skeletal abnormalities, including craniosynostosis [158] and osteoarthritis [113]. Mouse models recapitulate these findings, with *Ptch1* deletion causing elevated bone mass [98] and specific point mutations (e.g., *DL variant*) producing basal cell nevus syndrome‐like skeletal defects [158]. Human genetic studies further implicate 9q22.3 microdeletions encompassing *Ptch1* in severe skeletal malformations, including rib/spine deformities and intracranial calcifications [152, 159] while demonstrating that pathway overactivation underlies both tumorigenesis and disordered osteogenesis [56, 102, 160] (Table 2). These observations position Ptch1 as the critical regulator of Hh signalling intensity, where its disruption creates a shared etiological basis for seemingly disparate conditions ranging from cancer to skeletal dysplasia.

**TABLE 2:** Skeletal phenotypes associated with Hh signalling component deficiencies and related genetic disorders.

Gene	Mouse line	Phenotypes	Related skeletal disorders	Refs.
*PTCH1*	*Col2a1‐CreER*	Abnormal spine curvature, craniofacial disorder	Basal cell nevus syndrome 1 (OMIM: 109400) or Holoprosencephaly 7 (OMIM: 610828)	[102, 113, 152]
*SMO*	*LysM‐Cre; Osx‐Cre*	Bone loss, developmental defects and increased bone marrow adiposity	Curry‐Jones syndrome, somatic mosaic (OMIM: 601707) or Pallister‐Hall‐like syndrome (OMIM: 241800)	[59, 64, 98].
*GLI1*	*GLI1* ^*+/−*^	Decreased bone mineral density (BMD)	Polydactyly, postaxial, type A8 (OMIM: 618123) or Polydactyly, preaxial I (OMIM: 174400)	[161]
*GLI2*	Germline	Delayed ossification and deficiency of the medial portion of the frontal and parietal bones	Culler‐Jones syndrome (OMIM: 615849) or Holoprosencephaly 9 (OMIM: 610829)	[162]
*GLI3*	Germline	Postaxial polydactyly, skull and spinal malformation	Pallister‐Hall syndrome (OMIM: 146510); Greig cephalopolysyndactyly syndrome (OMIM: 175700); Polydactyly, postaxial, types A1 and B (OMIM: 174200); Polydactyly, preaxial, type IV (OMIM: 174700)	[61, 102, 152, 163, 164, 165]
*SUFU*	*Wnt1‐Cre or Dermo1‐Cre*	Postnatal death, loss of calvarial bones	Basal cell nevus syndrome 2 (OMIM: 620343)	[100]
*Ihh*	Germline	Shortening forelimbs and ribs of the endochondral skeleton/dwarfism	Acrocapitofemoral dysplasia (OMIM: 607778) or Brachydactyly, type A1 (OMIM: 112500)	[89]
*Shh*	Germline	Patterning defects A–P axis of the limb	Holoprosencephaly 3 (OMIM: 142945)	[166]

Aberrant *SMO* activation drives profound skeletal pathologies, with both gain‐ and loss‐of‐function perturbations disrupting bone homeostasis through distinct mechanisms. Constitutive *SMO* activity promotes osteoporosis and metabolic bone disorders [59, 64], while its conditional ablation paradoxically induces osteopenia [98], revealing context‐dependent roles in bone remodelling. GLI transcription factors emerge as critical nodal points, where mutations trigger severe developmental defects [25, 70, 130], *GLI3* dysfunction causes craniosynostosis and calvarial hyperossification [152], while *GLI2/GLI1* perturbations underlie syndromic conditions like Pallister‐Hall and Greig cephalopolysyndactyly [61, 151, 167, 168, 169, 170]. *GLI2* and *GLI3* mutations cause severe bone defects [129]. The pathway's precision is further highlighted by *SUFU* mutations, which disrupt anteroposterior patterning and calvarial morphogenesis [100] while causing polydactyly [86], often coincident with Ptch1‐related macrocephaly [171]. These findings collectively demonstrate that Hh signalling operates as an exquisitely tuned morphogenetic system, where component‐specific perturbations produce distinct but overlapping skeletal phenotypes through disruption of conserved regulatory circuits governing osteogenesis and chondrogenesis.

### 1.5 Targeted Therapies of Hh Signalling in Clinical Implications

The pharmacological modulation of Hh signalling presents a promising yet nuanced therapeutic approach for skeletal disorders, which requires precise balancing of pathway activation [48]. SMO inhibitors like GDC‐0449 (vismodegib) demonstrate efficacy in Hh‐driven malignancies such as medulloblastoma [172], while cyclopamine analogs suppress osteoblast activity and bone mineralisation [108], highlighting the pathway's dual roles in bone physiology. Conversely, Hh agonists (e.g., Hh‐Ag1.7, purmorphamine) promote fracture healing by stimulating chondrocyte and osteoblast proliferation [148] through SMO‐mediated upregulation of *BMP* and *Runx2* [173]. Endogenous regulators further complicate the therapeutic landscape: PXR antagonises Hh signalling via induction of suppressors (CDON/BOC/GAS1) [174], while Slitrk5 negatively regulates osteoblast differentiation through pathway inhibition [174]. These findings underscore the delicate equilibrium required for therapeutic targeting—where pathway activation enhances bone formation but risks pathological overgrowth, and inhibition preserves bone structure but may impair regeneration. Future strategies must therefore incorporate: (1) tissue‐specific delivery systems, (2) temporal control of pathway modulation and (3) combinatorial approaches with BMP/Wnt regulators to optimise skeletal outcomes while minimising off‐target effects.

Therapeutic targeting of canonical Hh signalling has emerged as a transformative strategy for skeletal disorders, with FDA‐approved SMO inhibitors (vismodegib, sonidegib) demonstrating remarkable efficacy in Gorlin syndrome and basal cell carcinomas by suppressing tumour proliferation through 90% pathway inhibition [175, 176, 177]. Beyond oncology, Hh modulation shows promise for McCune‐Albright syndrome [178] and osteosarcoma—where *GLI2* siRNA knockdown offers a precision approach [179]—while small molecules (SAG, cyclopamine) address calvarial defects [154] and vascular smooth muscle pathologies [180]. The discovery of novel regulators like GRP78/Shh in bone metastasis [179] and PI4KB inhibitor Pipinib in medulloblastoma [181] expands the therapeutic arsenal, complemented by epigenetic modulators (tazemetostat) that coordinately regulate Hh/Wnt/cAMP pathways [111]. Clinical insights reveal Hh's dual role: while SMO inhibitors impair fracture healing [106], pathway activation enhances bone modelling—a dichotomy necessitating tissue‐specific delivery systems. Current trials evaluating next‐generation inhibitors (BMS‐833923, saridegib) [175] underscore the pathway's clinical versatility, though uncharted side effects demand rigorous pharmacovigilance. These advances crystallise Hh signalling as a primary regulatory node amenable to pharmacological intervention across the skeletal disease spectrum (Table 3), from genetic disorders to metastatic cancer, through tailored modulation of its core components (SMO/GLI) and interacting pathways.

**TABLE 3:** Clinically approved drugs in treating Hh signalling disorders.

Therapeutic target	Hh signalling	Clinical implication	Outcomes	Refs.
Vismogib	SMO inhibitor	Tumour, Gorlin syndrome	90% decrease in the *Hh* signalling	[176]
Vismogib	SMO inhibitor	Metastatic solid tumours	Down‐modulation of GLI1 expression	[182]
GDC‐0449	SMO inhibitor	Medulloblastoma	Rapid tumour regression and decrease of symptoms	[172]
Sonidegib	SMO inhibitor	Basal cell carcinoma	> 90% and 80% response rates with sonidegib 200 and 800 mg respectively	[177, 183]
Sonidegib	SMO inhibitor	Medulloblastoma and basal cell carcinoma	Reduction in GLI1 mRNA expression	[184]
Pyrazole‐imidazole smoothib	SMO inhibitor	Medulloblastoma	Reduced expression of *Hh* target genes and suppression on medulloblastoma cells	[181]
Glasdegib	SMO inhibitor	Advanced solid tumours	> 80% downregulation of GLI1 expression	[185, 186]
TAK‐441	SMO inhibitor	Colorectal cancer, basal cell carcinoma, pancreatic cancer	Strong inhibition of GLI1 expression	[187]
Saridegib	SMO inhibitor	Medulloblastoma	Tumour reduction and significantly prolonged survival	[188]

## 2 Summary and Perspective

The Hh signalling pathway is a central regulatory hub in bone development, directing osteoblast differentiation through multilayered ligand‐receptor dynamics and GLI‐dependent transcriptional programming. This evolutionarily conserved system exhibits context‐dependent pleiotropy, integrating crosstalk with Wnt, BMP, FGF and PTHrP pathways to spatiotemporally coordinate skeletal morphogenesis and metabolic homeostasis. Recent advances underscore its dual regulatory role: while indispensable for osteoprogenitor specification, unchecked Hh activity drives pathological cascades, including heterotopic ossification, highlighting the necessity of stage‐specific pathway modulation.

Translational challenges now pivot on two frontiers: (1) designing spatially restricted Hh modulators that selectively inhibit heterotopic ossification while preserving physiological bone remodelling and (2) elucidating the skeletal sequelae of FDA‐approved SMO inhibitors (e.g., vismodegib/sonidegib), whose oncological efficacy in basal cell carcinoma is counterbalanced by underexplored osseous liabilities. Future breakthroughs demand systems‐level decoding of the logic underlying Hh signalling, particularly the GLI activator–repressor equilibrium and its crosstalk with osteogenic networks, as well as single‐cell omics‐guided target discovery and innovative drug‐release technologies. Therapeutic innovation will hinge on resolving these complexities, paving the way for precision interventions in genetic skeletal disorders and Hh‐engineered regenerative platforms. By marrying mechanistic insights from developmental biology with cutting‐edge musculoskeletal therapeutics, this paradigm promises to redefine the clinical management of bone diseases.

## Author Contributions

**Rohey Njie:** conceptualization (equal), data curation (equal), formal analysis (equal), visualization (equal), writing – original draft (equal), writing – review and editing (equal). **Shihan Xu:** validation (equal), visualization (equal). **Taofen Wu:** data curation (equal). **Jiashun Pi:** data curation (equal). **Sisi Lin:** validation (equal), visualization (equal). **Pengxiang Zhang:** data curation (equal). **Jiaqi Wang:** data curation (equal). **Qi Dai:** validation (equal), visualization (equal), writing – review and editing (equal). **Hui Shen:** validation (equal), visualization (equal), writing – review and editing (equal). **Nenghua Zhang:** supervision (equal), validation (equal), visualization (equal). **Guiqian Chen:** project administration (equal), supervision (equal), validation (equal), writing – original draft (equal), writing – review and editing (equal).

## Ethics Statement

The authors have nothing to report.

## Consent

All the authors agree to the current state of the manuscript to be submitted to the journal.

## Conflicts of Interest

The authors declare no conflicts of interest.

## Data Availability

All the data is within the manuscript.
